# Calcineurin Inhibitor Associated Nephrotoxicity in Kidney Transplantation—A Transplant Nephrologist's Perspective

**DOI:** 10.1111/apha.70047

**Published:** 2025-04-17

**Authors:** Carla M. Hansen, Sebastian Bachmann, Mingzhen Su, Klemens Budde, Mira Choi

**Affiliations:** ^1^ Department of Nephrology and Medical Intensive Care Charité—Universitätsmedizin Berlin, Corporate Member of Freie Universität Berlin and Humboldt Universität Zu Berlin Berlin Germany

**Keywords:** calcineurin inhibitor, kidney transplantation, toxicity

## Abstract

**Aim:**

Calcineurin inhibitors (CNIs) have revolutionized transplant medicine, improving allograft survival but posing challenges like calcineurin inhibitor‐induced nephrotoxicity (CNT). Acute CNT, often dose‐dependent, leads to vasoconstriction and acute kidney injury, with treatment focusing on CNI exposure reduction. Chronic CNT manifests as progressive allograft function decline, with challenges in distinguishing it from nonspecific allograft nephropathy.

**Methods:**

This narrative review provides a concise overview of the clinical management of CNT, covering acute and chronic CNT. We reviewed original articles, landmark papers, and meta‐analyses on CNT mitigation strategies, including CNI‐sparing approaches.

**Results:**

Preventive measures include co‐medications, CNI exposure monitoring, and CNI sparing strategies, such as reducing target trough levels and converting to mTOR inhibitors (mTORi) or belatacept. Despite improvements in graft function, challenges persist in demonstrating significant differences in allograft survival with CNI‐sparing regimens. The paradigm shift from chronic CNT as the main cause of chronic allograft nephropathy toward rather immunologic triggered injuries and/or comorbidities as relevant contributors to allograft deterioration over time must be kept in mind.

**Conclusion:**

CNIs have significantly improved kidney transplant outcomes, but their associated nephrotoxicity necessitates mitigation strategies. The decision to implement such regimens is always an individual choice balancing against the risk of immunologic injuries. Further long‐term studies are needed to optimize immunosuppressive approaches and refine CNT management.

**Abbreviations**
AKI: acute kidney injury
CCB: calcium channel blocker
CMV: cytomegalovirus
CNI: calcineurin inhibitor
CNT: calcineurin inhibitor‐induced nephrotoxicity
CTLA4: T‐lymphocyte‐associated antigen 4
dnDSA: de novo donor‐specific antibodies
EBV: Epstein–Barr virus
FDA: Food and Drug Administration
GFR: glomerular filtration rate
HUS: hemolytic uremic syndrome
MMF: mycophenolate mofetil
MPA: mycophenolic acid
mTORi: mammalian target of rapamycin inhibitor
NODAT: new‐onset diabetes after transplantation
PTLD: post‐transplant lymphoproliferative disorder
RAAS: renin‐angiotensin‐aldosterone system
RCT: randomized controlled trial

## 1 Introduction

Over the past four decades, calcineurin inhibitors (CNIs) have played a pivotal role in immunosuppressive regimens for post‐kidney transplantation care, significantly reducing acute rejection rates and improving allograft survival. However, their use is associated with relevant side effects, including calcineurin inhibitor‐induced nephrotoxicity (CNT), as well as cardiovascular and metabolic complications [1, 2]. As of the present day, international guidelines for the diagnosis and management of CNT are limited [3]. This narrative review aims to provide an overview on CNT, covering landmark papers, systematic reviews, and meta‐analyses relevant for transplant nephrologists in their clinical practice.

Cyclosporine was discovered in 1969 and has been utilized since 1983 for the prophylaxis of rejection in transplant medicine [4]. This development represented a breakthrough in the field of organ transplantation; however, it was soon followed by the identification of adverse effects [5]. Tacrolimus received FDA approval in 1994 [6]. Both substances exert their immunosuppressive effects by interfering with the phosphatase calcineurin, leading to impaired T cell activation and reduced expression of interleukin‐2 and other cytokines [7]. Azathioprine was introduced in the 1960s and was used in combination with cyclosporine and prednisolone until the development of the antimetabolite immunosuppressants mycophenolic acid (MPA) or its ester pro‐drug, mycophenolate mofetil (MMF) in 1995 [8, 9]. Both substances act by inhibiting inosine monophosphate dehydrogenase, which blocks de novo purine synthesis in T and B lymphocytes, leading to reduced lymphocyte proliferation [10]. Due to their potent immunosuppressive effect, the standard post‐kidney transplantation immunosuppressive protocol has evolved since their introduction. Today, it typically includes a combination of CNIs, the antimetabolite MMF or MPA, and a corticosteroid to prevent allograft rejection [11, 12]. Tacrolimus has emerged as the preferred CNI due to its superior efficacy in reducing acute rejection rates [13, 14]. However, both CNIs are associated with side effects such as hypertension, new‐onset diabetes after transplantation (NODAT), dyslipidemia, hyperuricemia, electrolyte disorders, gastrointestinal side effects, nephrotoxicity, neurotoxicity, hair loss or hypertrichosis, and gingival hyperplasia, with some of the side effects specific to each CNI. Neurotoxicity, such as headache, tremor, seizures, and insomnia, and NODAT are more marked with tacrolimus, while hypertrichosis and gingival hyperplasia are more common in patients receiving cyclosporine [15, 16]. Further alternative immunosuppressive strategies emerged with the use of mammalian target of rapamycin inhibitors (mTORi) sirolimus and everolimus. Both substances received FDA approval in 1999 (sirolimus) and 2009 (everolimus), respectively [17, 18]. Lately, another option followed with the introduction of belatacept, a fusion protein of immunoglobulin IgG1 and T‐lymphocyte‐associated antigen 4 (CTLA4), approved in 2011 for its de novo use in kidney transplantation [19, 20].

Despite their pivotal role in preventing rejection, CNIs pose significant challenges due to their adverse effects, particularly nephrotoxicity. Thus, a patient‐tailored immunosuppressive regimen is mandatory to balance the risk of rejection, toxicity, and complications, for example, infections, to improve short‐ and long‐term clinical outcomes and graft outcomes.

## 2 Acute CNI‐Induced Nephrotoxicity

Acute CNT is most often dose‐dependent and typically occurs at supratherapeutic CNI serum trough levels exceeding 20 ng/mL for tacrolimus and 400 ng/mL for cyclosporine; on the other hand, it can occur at any drug level [21, 22, 23].

Acute CNT is attributed to the dysregulation of vasoactive components. The renin‐angiotensin‐aldosterone system (RAAS) and other vasoconstrictive substances (e.g., thromboxane and endothelin) may be activated, whereas concentrations of vasodilating substances (e.g., nitric oxide, prostaglandin E2, and prostacyclin) may be decreased [24]. This may critically alter allograft hemodynamics and, in particular, lead to vasoconstriction of the afferent arteriole, causing acute kidney injury (AKI) [25, 26].

Histopathological examination reveals degeneration and hyalinosis of smooth muscle cells in afferent arterioles and tubular damage characterized by isometric vacuolization. Clinical symptoms associated with acute CNT are consistent with those of CNI intoxication and may include headache, altered mental status, tremor, and elevated blood pressure [27]. Laboratory findings include elevation in serum creatinine levels unexplained by other causes and supratherapeutic trough levels of CNIs.

The key therapeutic approach in managing acute CNT is reducing CNI trough levels and addressing other factors potentially contributing to AKI.

CNIs have been associated with the de novo manifestation of thrombotic microangiopathy (TMA), which occurs in approximately 0.8%–3.2% of kidney transplant recipients [28, 29]. While some patients exhibit systemic hemolytic uremic syndrome (HUS), others present with localized renal TMA in allograft biopsies [30]. Thus, diagnosis might be challenging if TMA is only present in the kidney biopsy without laboratory findings such as hemolytic anemia, complement dysregulation, or the presence of schistocytes. Worth noting, other risk factors than the use of CNIs have been associated with de novo TMA, such as infections, antibody‐mediated rejection, and previously undetected HUS as the primary kidney disease, and the use of mTORi [31, 32, 33, 34, 35, 36]. Addressing the underlying cause is key for successful treatment and requires timely differential diagnosis. In the case of suspected CNI‐associated atypical HUS, CNI withdrawal is usually the first line of treatment [37]. In severe cases with AKI, thrombocytopenia, and/or systemic symptoms, plasmapheresis and the use of eculizumab, a monoclonal antibody that inhibits the complement protein C5, may be considered and is particularly approved for the treatment of complement‐mediated TMA [37, 38, 39]. Predictors of response to eculizumab for patients with CNI‐induced de novo TMA are scarce, as only anecdotal case reports are available so far [38, 40, 41, 42]. Therefore, clinical data are insufficient to provide reliable recommendations for the use of eculizumab in patients with CNI‐induced de novo TMA after kidney transplantation.

## 3 Chronic CNI‐Induced Nephrotoxicity

**FIGURE 1:**
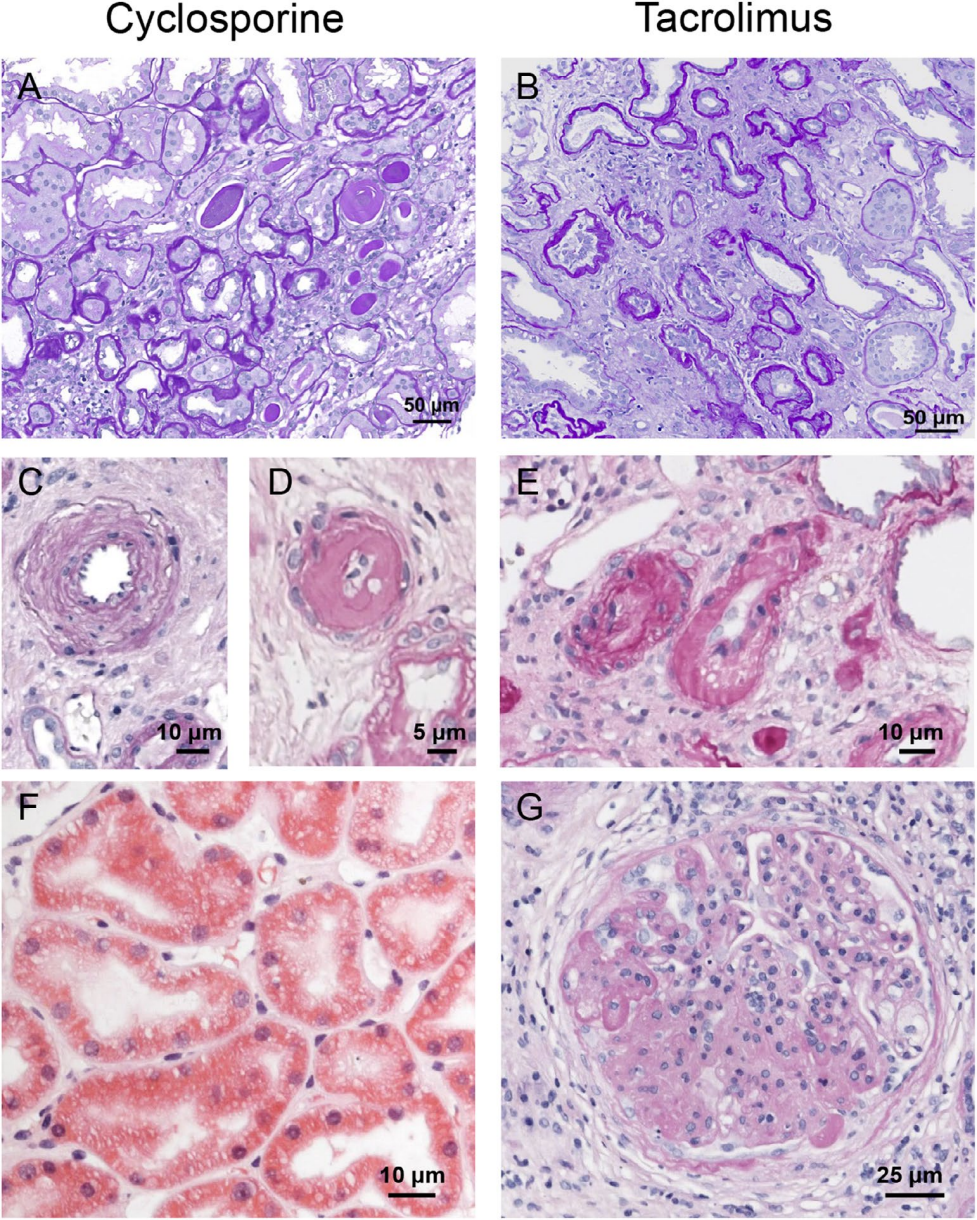
Histopathology of CNT in human kidney biopsies from cyclosporine and tacrolimus‐based treatment regimens. (A, B) Interstitial fibrosis/tubular atrophy; note hyaline tubular casts in (A). (C–E) Arteriolar wall changes. Media hypertrophy and hyalinosis in Cyclosporine (C, D), luminal narrowing and perivascular inflammation in tacrolimus‐based treatment regimen (E). (F) Tubular vacuolization in proximal tubule. (G) Focal segmental glomerulosclerosis with focal adhesions to capsule. PAS staining (A–E, G), hematoxylin eosin (F); bars indicate magnification. Courtesy of Kerstin Amann, Marie‐Christine Heinrich.

Chronic CNT manifests as a progressive decline in allograft function, characterized by a gradual decrease in glomerular filtration rate (GFR) over time. The underlying mechanisms involve alterations in renal hemodynamics resulting in ischemia, as well as direct toxic effects on tubular epithelial cells. The parenchymal damage associated with chronic CNT is largely irreversible [43]. Histopathological analysis typically reveals hyaline arteriolopathy, tubular atrophy, and striped interstitial fibrosis [44]. Typical damage patterns are illustrated in Figure 1. Distinct histopathological profiles may be seen with tacrolimus and cyclosporine‐based immunosuppression regimens [45]. Compared to cyclosporine‐based regimens, tacrolimus‐based regimens have been associated with less early CNT and fewer rejection episodes, but comparable chronic arteriolar toxicity [45, 46]. Biopsy specimens with up to 10 years of follow‐up showed a greater incidence of striped fibrosis and tubular microcalcification in the cyclosporine “era,” while the prevalence of moderate arteriolar hyalinosis was similar in both CNIs [46]. The severity of biopsy findings correlates with the dose and duration of CNI use [46].

While chronic CNT has been postulated as one major cause of chronic allograft damage and allograft loss for many years, since the early 2000s a paradigm shift pointed towards the immunologic injury as the main unrecognized reason for chronic allograft deterioration. A 2003 prospective study examining protocol biopsies of kidney allografts in patients with type 1 diabetes undergoing combined kidney and pancreas transplantation revealed that the prevalence of CNI‐related histopathological findings steadily increased over time. These findings were present in over half of renal allograft biopsies at the 3‐year mark and in all biopsies by the 10‐year post‐transplantation mark [47]. However, despite marked histological signs of chronic CNT under prolonged high cyclosporine exposure (average trough level of 204 ng/mL over 10 years), 10‐year outcomes showed a graft survival of 84.4% and a mean serum creatinine level of 1.6 mg/dL. Subsequent research has indicated that histological lesions which had been attributed to chronic CNT may be nonspecific and have multifactorial underlying causes [48, 49, 50]. This has also been highlighted in allograft biopsies from both therapy compliant and noncompliant patients, which demonstrated similar histological patterns of interstitial leucocyte infiltrate, interstitial fibrosis, and tubular atrophy, with comparable degrees of arteriolar hyalinosis between groups. This demonstrated that arteriolar hyalinosis developed in kidney transplant recipients regardless of cyclosporine use and was independently associated with an increased risk of graft loss [51]. Snanoudj et al. demonstrated that arteriolar hyalinosis developed in kidney transplant recipients regardless of cyclosporine use and was independently associated with an increased risk of graft loss [52]. Thus, distinguishing between chronic CNT and chronic, nonspecific allograft nephropathy remains a challenge both clinically and histologically [53, 54]. Immune and nonimmune mechanisms contribute to kidney allograft injury over time, leading to chronic interstitial fibrosis and tubular atrophy [47, 50, 55]. Mayrdorfer et al. analyzed the relative contributions of CNT to graft loss and found that CNT was the primary cause in fewer than 1% of graft loss cases, while it was considered a secondary cause in 20.5% of cases [56]. Moreover, they demonstrated that approximately one third of primary causes for allograft failure were attributed to T cell‐mediated rejection and/or antibody‐mediated rejection. Therefore, immunosuppression requires balancing rejection prophylaxis with the risk of overimmunosuppression and side effects, necessitating a careful evaluation of benefits, risks, and alternative treatable diagnoses. The introduction of molecular studies, for example, the molecular microscope diagnostic system, and the implementation of biomarkers will help gain more insights into the histopathologic features and multifaceted causes of allograft damage and may pave the way towards a personalized immunosuppression [57, 58].

Based on current evidence, we consider chronic CNT as one contributing factor to chronic allograft nephropathy. However, we strongly advocate for critical reassessment of this diagnosis, with consideration given to alternative, potentially treatable differential diagnoses.

## 4 Preventive Measures of CNI‐Induced Nephrotoxicity

### 4.1 Co‐Medication

The use of various co‐medications has been studied as a strategy for the mitigation of CNT and improvement of allograft survival.

Calcium channel blockers (CCBs) have been studied for their ability to induce vasodilation and counteract the vasoconstriction caused by CNIs as well as by other vasoconstrictors—such as endothelin and thromboxane—whose production may be affected by cyclosporine [59, 60]. Dihydropyridine CCBs (e.g., nifedipine, amlodipine) selectively relax vascular smooth muscle, leading to vasodilation and blood pressure reduction, while non‐dihydropyridine agents (e.g., verapamil, diltiazem) primarily affect the heart, decreasing heart rate and contractility. While both classes are substrates of CYP3A4, non‐dihydropyridine CCBs are also inhibitors of CYP3A4 and, therefore, cause more drug–drug interactions [61]. Used as a co‐treatment regimen, nitrendipine was associated with a higher GFR in renal‐transplant patients at 24 months [62]. Diltiazem demonstrated a cyclosporine‐sparing effect, requiring lower cyclosporine doses to maintain therapeutic levels [61]. The co‐administration of CCBs was linked to reduced rejection severity, particularly vascular rejection, and lower primary nonfunction rates [63] and better allograft function at 2 years [64]. In contrast, Weinrauch et al. retrospectively investigated the impact of CCB intake versus none in 4110 kidney transplant recipients between 2002 and 2007, followed until 2010. They did not find statistically significant differences in incidence rates of cardiovascular, non‐cardiovascular, and all‐cause mortality between patients taking CCB medications versus none [65]. Furthermore, a 3‐year randomized study found no GFR or proteinuria benefits with dihydropyridine CCBs [66]. A meta‐analysis found that CCBs reduced the risk of graft loss by 42%, increased GFR by 3.08 mL/min, and lowered blood pressure [67]. The European Society of Hypertension therefore recommends using CCBs as the preferred anti‐hypertensive agent in the early post‐transplantation period [68].

In summary, CCBs, especially diltiazem and nifedipine, may reduce cyclosporine needs and improve graft function, but evidence on long‐term benefits is inconsistent and broader clinical advantages remain uncertain [63, 65, 69].

RAAS activation has also been shown to play a role in the pathophysiology of chronic CNT [70]. Despite promising findings from animal studies, randomized controlled trials (RCTs) evaluating the use of angiotensin‐converting enzyme inhibitors, angiotensin‐II receptor blockers, and aldosterone receptor antagonists have not shown significant benefits [71, 72]. Recent results from the SPIREN trial—a randomized, placebo‐controlled, double‐blind study involving 188 kidney transplant recipients randomized to receive either spironolactone or placebo and followed over three years—demonstrated that spironolactone treatment led to an acute decline in measured GFR (up to −7.6 mL/min compared with placebo) and reduced 24‐h proteinuria after one year. However, the overall GFR slope during the follow‐up period was similar between the spironolactone and placebo groups [72]. In the same line, protocol biopsies revealed no significant differences in Banff scores for arteriolar hyalinosis or interstitial fibrosis [66].

The overall long‐term impact of co‐medications on CNT remains unclear due to conflicting data, short observation periods, and variable endpoints lacking histological confirmation. The strongest evidence supports the use of CCBs for treating hypertension after kidney transplantation. However, the effect of co‐medications on CNT appears limited and should not be overstated.

### 4.2 CNI Exposure

CNI exposure is influenced by drug dose, serum concentration (trough levels or area under the curve [AUC]), and treatment duration. High CNI exposure increases the risk of nephrotoxicity, while low exposure may lead to inadequate immunosuppression and rejection [73, 74]. Due to the narrow therapeutic windows and high inter‐individual variability in dose‐to‐serum concentration ratios, therapeutic drug monitoring of CNI trough levels has become standard practice [75, 76]. Notably, more so for cyclosporine, the typically measured trough level (serum concentration measured right before the next dose administration, C_0_) seems to correlate less with total dose compared to measuring the area under the time‐concentration curve (AUC_0to12_) [77].

Effective serum concentrations of cyclosporine and tacrolimus are influenced by the expression and activity of metabolizing enzymes CYP3A4 and CYP3A5 and the multidrug efflux pump P‐glycoprotein [78, 79]. Polymorphisms in CYP3A4 and CYP3A5, as well as concomitant use of medications that interact with CYP3A4, CYP3A5, or P‐glycoprotein, can result in both increased and decreased serum levels of CNIs. Consequently, genotyping for CYP3A418B and CYP3A53 before kidney transplantation has shown promising results in determining appropriate CNI dosing but is not yet part of routine clinical practice [80, 81, 82]. To avoid potential interactions with CNIs, it is crucial to check patients' medication and avoid pharmaceuticals with potential interactions, if possible, or make respective dose adjustments. Additionally, episodes of diarrhea can increase serum concentrations of tacrolimus, which may be attributed to altered intestinal CYP3A activity and decreased drug efflux by intestinal P‐glycoprotein [83, 84].

In conclusion, optimizing CNI exposure requires therapeutic drug monitoring due to high inter‐individual variability. While pharmacogenetic testing seems promising, it is not yet routine. Managing drug interactions and patient‐specific factors, such as gastrointestinal disturbances, is crucial for maintaining stable CNI levels and minimizing nephrotoxicity.

## 5 CNI Minimization Strategies

### 5.1 Reduction of CNI

The introduction of MMF in 1995 had a significant impact on the ability to reduce CNI trough levels by providing an alternative to azathioprin while maintaining adequate immunosuppression [85]. Reducing the target trough level (T_0_) of CNIs has been investigated in various RCTs [86, 87].

The ELITE‐Symphony study, a landmark trial in the field, investigated four different MMF‐ and steroid‐based immunosuppressive regimens with CNI minimization or avoidance in a 12‐month prospective trial [88]. At the time of kidney transplantation, patients were randomized to receive daclizumab induction, MMF, and corticosteroids in combination with either standard‐dose cyclosporine, low‐dose cyclosporine, low‐dose tacrolimus, or low‐dose sirolimus. At 12 months post‐transplantation, GFR was the primary endpoint and was significantly higher in the low‐dose tacrolimus group compared to the other study groups. Moreover, the study demonstrated a lower rate of biopsy‐proven acute rejection in patients receiving low‐dose tacrolimus (12.3%) than in those receiving standard‐dose cyclosporine (25.8%) or low‐dose cyclosporine (24.0%). The cohorts with a lower CNI target dose regimen had a significantly better allograft survival compared to the standard‐dose cyclosporine group. Before the ELITE‐Symphony trial, Vincenti et al. investigated the complete avoidance of CNI using daclizumab induction with MMF and steroids only [89]. Despite a good allograft survival of 97% after 12 months, rejection rates were high (53%). Thus, further studies considered the use of low‐dose CNI or alternative concomitant immunosuppressive strategies. Along the same line, systematic reviews and meta‐analyses have shown better clinical outcomes, including improved allograft function with reduced CNI exposure; however, without significant differences regarding mortality [90, 91]. The timing of reduction of CNI exposure also seemed to correlate with outcomes, as a subgroup analysis of a Cochrane meta‐analysis found. The review indicated that early CNI withdrawal or tapering (within the first few months post‐transplant) was associated with a higher risk of acute rejection, whereas delaying the intervention (typically after one year) tended to improve renal outcomes with a lower rejection risk [91].

Reducing CNI exposure has shown benefits in preserving allograft function while complete CNI avoidance increases the risk of rejection. Thus, a cautious, delayed reduction strategy appears to optimize renal outcomes without compromising graft survival.

### 5.2 Conversion From CNI to MTOR Inhibitor

MTORis have been used in de novo kidney transplantation to reduce or omit CNI or as an option to convert from, for example, CNI or MMF. Several trials and meta‐analyses comparing mTORi‐based and CNI‐based immunosuppression post‐kidney transplantation found a higher GFR, increased acute rejection rates, fewer Cytomegalovirus (CMV) infections, reduced malignancy, and more lymphoceles in the mTORi group [88, 91, 92, 93, 94]. De Fijter et al. investigated early conversion to mTORi 10–14 days post‐transplant versus continued CNI. At 12 months, the groups showed similar GFR; however, in the mTORi group a higher incidence of biopsy‐proven acute rejection and a potential increase in de novo donor‐specific antibodies (dnDSA) was observed [95]. In a related single‐center study, 23% (14/61) of patients converted from cyclosporine to mTORi between 3‐ and 4.5‐months post‐transplant developed dnDSAs, compared with 11% (7/65) patients who remained on cyclosporine [96].

Late conversion, defined as conversion from CNI to mTORi after more than 6 months post‐transplant, showed reduced malignancy rates and improved GFR with no significant differences in graft loss, chronic allograft nephropathy, and mortality [97, 98]. Common side effects included increased infection rates, myelosuppression, gastrointestinal symptoms, impaired wound healing, dyslipidemia, post‐transplant diabetes mellitus, pneumonitis, and dermatologic disorders.

Notably, discontinuation of medication secondary to adverse events was more common in patients on mTORi than in patients on CNI [95, 98].

In summary, conversion to an mTORi‐based regimen may be appropriate for selected patients, such as those experiencing severe CNI‐associated adverse effects (e.g., neurotoxicity or CNT). Conversely, patients with a reduced GFR and/or significant proteinuria are unlikely to benefit from transitioning from CNIs to mTORis [99].

### 5.3 Conversion to Belatacept

Administered intravenously, belatacept is considered an alternative to CNIs when combined with an antimetabolite agent and corticosteroid [20]. Side effects include infections such as CMV infections, gastrointestinal symptoms, cytopenia, and the increased risk for post‐transplant lymphoproliferative disorder (PTLD) particularly in EBV‐seronegative patients. As a result, it is contraindicated in patients with negative or unknown EBV status [100].

Various studies and meta‐analyses have evaluated belatacept‐based regimens [101, 102, 103, 104]. They found no significant differences in kidney allograft survival and mortality. However, belatacept‐based regimens were associated with lower rates of NODAT, dyslipidemia, hypertension, and chronic allograft nephropathy [105, 106]. It also reduced the occurrence of dnDSA but resulted in higher rates of acute rejection in some studies [107].

The BENEFIT‐ and the BENEFIT‐EXT study are the two RCTs with the longest observational period (up to seven years) published so far. They both compared a belatacept‐based to a cyclosporine‐based primary immunosuppression regimen. The BENEFIT trial included participants receiving kidneys from living or standard criteria deceased donors. In this trial, one year after transplantation, the belatacept group demonstrated significantly lower rates of graft loss and mortality, along with markedly improved GFR, despite a higher incidence of acute rejection (17% with belatacept versus 7% with cyclosporine) [101, 108].

In the BENEFIT‐EXT trial, only patients receiving extended criterion donor kidneys were included, that is, both participants and donors were older and had more comorbidities. In this RCT, even though the study design was otherwise very similar to the BENEFIT trial, no statistically significant difference for graft loss and death was observed, but an improvement in allograft function was observed [108]. Moreover, in both studies, significantly less dnDSA were observed in the belatacept group compared to the cyclosporine group over seven years [109]. Nonetheless, some trials revealed significantly higher rejection rates in belatacept‐treated patients compared to patients receiving cyclosporine [110].

Trials specifically looking into the conversion of the immunosuppression regimen to belatacept later than 6 months after transplantation (i.e., during the maintenance period of the immunosuppression regimen) found comparable rates of mortality and graft loss, for example, as demonstrated by Grinyo et al. 12 months after conversion from a CNI‐based immunosuppressive therapy to belatacept [111]. In their long‐term extension phase II study up to two years, patients converted from CNI to belatacept showed a higher mean GFR increase than patients with CNI maintenance [112]. Brakemeier et al. retrospectively analyzed 79 patients converted from CNI to belatacept after an average of 69 months, mainly due to biopsy‐proven CNT, and observed a significant improvement in GFR at 12 months post‐conversion [113]. In a randomized phase III trial, kidney transplant recipients were randomized to belatacept conversion versus CNI continuation. A similar survival rate and a superior 24‐month GFR, as well as a lower incidence of dnDSA, despite an increased incidence of biopsy‐proven acute rejection (1% versus 7%), were observed [114]. A recent study reported long‐term outcomes seven years after conversion from CNI to belatacept versus CNI continuation in 243 patients, each with comparable rates of death, antibody‐mediated rejection, and T cell‐mediated rejection [115].

Conversion from a CNI‐based immunosuppression to belatacept is also an alternative treatment strategy in patients with concerns to continue with a CNI‐based regimen or to ensure better therapy adherence [116, 117].

In conclusion, belatacept offers a viable alternative to CNIs in kidney transplantation, demonstrating benefits such as improved allograft function, lower rates of NODAT, dyslipidemia, and dnDSA formation. However, its use is limited by an increased risk of acute rejection and PTLD, particularly in EBV‐seronegative patients. Further research is needed to refine patient selection and optimize immunosuppressive strategies to balance rejection risk with long‐term graft preservation.

Table 1 presents a summary of findings from meta‐analyses that examined various CNI‐sparing immunosuppressive regimens after kidney transplantation. We performed a systematic PubMed search using the search term (“calcineurin inhibitor*”[*ti*] *OR tacrolimus*[*ti*] OR ciclosporin*[*ti*] OR cyclosporin*[*ti*]) *NOT steroid*[*ti*] *AND* (*toxicity*[*tiab*] *OR nephrotoxicity*[*tiab*] *OR withdrawal*[*tiab*] *OR tapering*[*tiab*] *OR reduction*[*tiab*] *OR sparing*[*tiab*] *OR avoidance*[*tiab*]) *AND* “*kidney transplantation*”[*MAJR*], which led us to identify 13 meta‐analyses [90, 91, 104, 118, 119, 120, 121, 122, 123, 124, 125]. We excluded two that did not primarily address CNI‐sparing following kidney transplantation and analyzed the remaining ones for outcomes. All publications included RCTs except for one that also included non‐RCTs [124, 125]. Notably, some overlap in original study data and patients across the listed meta‐analyses may exist. Therefore, any quantitative analysis would not provide an accurate or meaningful synthesis. However, Table 1 offers an overview of key research guiding clinical practice in the absence of international guidelines. While the table does not present pooled results, it summarizes important findings that are currently guiding clinical decision‐making in the field.

**TABLE 1:** Overview on findings from meta‐analyses that examined various CNI‐sparing immunosuppressive regimens in kidney transplantation.

First author	Publication year	Intervention^a^	Observation periods	Number of trials included^b^	Result mortality	Result graft loss	Result rejection rates	Result GFR
Karpe KM	2017	CNI withdrawal (avoidance or late withdrawal)	6–24 months; endpoint mortality: 9–20 years	*n* = 17	RR 1.09, 95% CI 0.96–1.24	RR 0.85, 95% CI 0.74–0.98	RR 2.54, 95% CI 1.56–4.12	MD 3.56, 95% CI −1.25 to 8.25
		Low dose CNI versus standard dose CNI		*n* = 18	RR 0.79, 95% CI 0.50–1.27	RR 0.75, 95% CI 0.55–1.02	RR 0.87, 95% CI 0.76–1.01	MD 4.10, 95% CI 2.07–6.12
		CNI withdrawal or avoidance with mTOR‐I		*n* = 29	RR 0.96, 95% CI 0.68–1.36	RR 0.94, 95% CI 0.75–1.19	RR 1.43, 95% CI 1.15–1.78	MD 5.29, 95% CI 2.08–8.51
		Low dose CNI and mTORi		*n* = 14	RR 1.16, 95% CI 0.71–1.90	RR 0.67, 95% CI 0.45–1.01	RR 1.13, 95% CI 0.91–1.40	MD 6.24, 95% CI 3.28–9.19
Liu J	2017	CNI conversion to Everolimus	12 months	*n* = 15	RR 0.70, 95% CI 0.22–2,18	RR 1.43, 95% CI 0.44–4.68	RR 1.82, 95% CI 1.11–2.99	MD 5.36, 95% CI 2.32–8.39
			5 years		RR 0.70, 95% CI 0.29–2,46	RR 1.10, 95% CI 0.51–5.70	RR 1.85, 95% CI 0.94–3.65	MD 6.50, 95% CI 2.38–10.63
Sawinski D	2016	CNI minimization	12 months with variability	*n* = 36	RR 0.91, 95% CI: 0.72–1.14	RR 0.76, 95% CI: 0.61–0.94	RR 0.84, 95% CI: 0.75–0.95	MD 0.32, 95% CI: 0.22–0.41
		CNI conversion to mTORi or Belatacept		*n* = 23	No difference	No difference longterm	More acute rejections	GFR higher
		CNI withdrawal		*n* = 13	No difference	No difference	More acute rejections	GFR higher
		CNI avoidance		*n* = 9	No difference	No difference	More acute rejections	GFR higher
Talawila N	2015	CNI minimization/withdrawal + Belatacept	12 months	*n* = 6	OR 1.46, 95% CI 0.61–3.5	OR 1.20, 95% CI 0.75–1.92	OR 1.65, 95% CI 0.83–3.30	MD 11.7, 95% CI 0.09–23.35
			24 months	*n* = 2	OR 1.67, 95% CI 0.99–2.81	OR 1.03, 95% CI 0.65–1.64	OR 1.77, 95% CI 0.83–3.79	MD 13.7, 95% CI 6.34–21.10
Bai H	2015	CNI withdrawal	6–24 months	*n* = 7	RR 0.99, 95%, CI 0.98–1.01	RR 1.00, 95% CI 0.98–1.02	RR 1.64, 95% CI 1.19–2.27	MD 9.50, 95% CI 2.96–16.03
Su L	2014	CNI sparing + Everolimus	12–24 months	*n* = 7	RR 1.07, 95% CI 0.73–1.58^c^		RR 2.51, 95% CI 1.63–3.87	SC MD −9.94 μmol/L, 95% CI −16.66–(−3.22 μmol/L)
Yan HL	2014	CNI avoidance	12 months	*n* = 16	OR 0.90, 95% CI 0.47–1.69	OR 0.73, 95% CI 0.47–1.12	OR 1.74, 95% CI 1.08–2.81	MD 5.31, 95% CI 0.30–10.31
			24 months		OR 0.91, 95% CI 0.44–1.87	OR 0.87, 95% CI 0.48–1.57, *p* = 0.64	OR 0.92, 95% CI 0.33–2.51	MD 13.96, 95% CI 7.32–20.60
		CNI withdrawal	12 months	*n* = 11	OR 0.74, 95% CI 0.41–1.32	OR 0.69, 95% CI 0.46–1.03	OR 1.78, 95% CI 1.35–2.34	MD 7.03, 95% CI 4.84–9.23
			24 months		OR 0.73, 95% CI 0.42–1.29	OR 0.81, 95% CI 0.56–1.16	OR 2.42, 95% CI 1.01–5.82	
Sharif A	2011	CNI avoidance	6–36 months	*n* = 32	OR 0.92, 95% CI 0.76–1.11	OR 1.05, % CI 0.85–1.29	OR 1.24, 95% CI 1.01–1.53	MD 5.31, 95% CI 2.82–7.81
		CNI minimization		*n* = 17		OR 0.73, 95% CI 0.58–0.92	OR 0.99, 95% CI 0.76–1.28	MD 3.44, 95% CI 1.21–5.68
		Delayed introduction of CNI		*n* = 10		OR 1.04 95% CI 0.75–1.44	OR 1.00, 95% CI 0.67–1.50	MD 2.83, 95% CI 0.09–5.76
Moore J	2009	CNI minimization and elimination	6–24 months	*n* = 19	OR 1.08, 95% CI 0.68–1.72	OR 0.72, 95% CI 0.52–1.01	Strong hetero‐genity	MD 4.4, 95% CI 2.9–5.9
Kasiske BL	2000	Cyclosporine withdrawal	12–93 months	*n* = 13		RR 1.06, 95% CI 0.82–1.29	Pooled difference 0.11, 95% CI 0.07–0.15	
Kasiske BL	1993	Elective cyclosporine withdrawal	12–36 months	*n* = 17^d^	Weighted difference in deaths per patient per year, −0.005, 95% CI −0.016 to 0.006	Weighted difference in grafts lost per patient per year, −0.009, 95% CI −0.022 to 0.004	Weighted difference in episodes per patient, 0.126, 95% CI 0.085 to 0.167	Mean SC level standard group 163 ± 26 μmol/L vs. cyclosporine withdrawal group 144 ± 25 μmol/L

*Note:* green = intervention had a favorable impact, red = intervention had an unfavorable impact, gray = intervention did not show to have a statistically significant effect compared to control group.

Abbreviations: CI, confidence interval; CNI, calcineurin inhibitor; GFR, glomerular filtration rate in mL/min/1.73 m^2^; MD, mean difference; mTORi, mammalian target of rapamycin inhibitor; OR, odds ratio; RR, relative risk; SC, serum creatinine; vs., versus.

^a^ Versus standard dose CNI.

^b^ Number of trials analyzed for each outcome varied.

^c^ Death and graft loss = categorized as event.

^d^ *n* = 10 NRCT.

## 6 Conclusion

CNIs have significantly improved outcomes in kidney transplantation. Nevertheless, their associated CNT and adverse metabolic and cardiovascular effects, such as hypertension, dyslipidemia, and NODAT, underline the need to use alternative approaches [126, 127]. Notably, only in very few cases, CNT is the leading cause of graft loss, while being one of several contributing factors in over 20% [56].

The clinical management of CNT after kidney transplantation is based on physicians' expertise and individual patient considerations. Acute CNT necessitates a reduction in CNI exposure, while accurately diagnosing and managing chronic CNT remains challenging due to the lack of highly specific clinical and histological features. Various RCTs and meta‐analyses have investigated co‐medication, CNI avoidance, reduction, withdrawal, and conversion to alternative immunosuppressive agents [73, 91, 97, 106]. Importantly, most RCTs have primarily focused on CNI‐free or reduced CNI exposure regimens. There is a lack of studies that prospectively evaluate treatment options after the diagnosis of chronic CNT.

The scientific evidence from RCTs and meta‐analyses investigating CNI sparing regimens predominantly demonstrates an improvement in GFR but no significant differences in allograft survival. Expectedly, the reduction in CNI exposure results in an improvement in GFR due to the hemodynamic impact of CNIs on renal blood flow. Thus, an increase in GFR alone should not be considered a definitive marker of long‐term graft benefit without concurrent evidence of improved allograft survival. The implication of the reported increase in the incidence of acute rejection following CNI reduction or withdrawal remains of critical concern in regard to long‐term graft survival. Acute rejection episodes have been associated with an increased risk of chronic allograft dysfunction and reduced graft survival, particularly when rejection is severe or recurrent. Further long‐term follow‐up studies are needed to determine whether the benefits of CNI reduction outweigh the potential risks associated with rejection episodes [90, 91]. Belatacept‐based regimens have shown favorable outcomes in the BENEFIT trial. Interestingly, these effects were not significant in the BENEFIT‐EXT trial, which specifically included patients receiving kidneys from extended criteria donors [108].

A retrospective study looking into late conversion (mean 11.9 years after transplantation) to belatacept in kidney transplant recipients with biopsy‐proven CNT found, again, an improvement in GFR while allograft and patient survival remained unexamined [128].

To date, only a few RCTs evaluated CNI‐sparing regimens in long‐term observation periods [129]. To address the question of whether CNI‐sparing regimens can improve long‐term outcomes, further studies with extended observation periods are required. Future research should also focus on identifying patients who may benefit most from CNI reduction while minimizing rejection risk, potentially through biomarker‐guided or individualized immunosuppressive strategies.

In conclusion, while triple immunosuppressive therapy remains the standard of care, the decision to implement a CNI‐free regimen, including its modality and timing, is highly complex and must be individualized based on patient‐specific risk factors and clinical parameters.

Further research, including studies with long‐term outcomes and large sample sizes, is necessary to enhance our understanding in this field.

## Author Contributions

**Carla M. Hansen:** conceptualization, data curation, writing – original draft, writing – review and editing, investigation, methodology, project administration, supervision, formal analysis. **Sebastian Bachmann:** writing – review and editing, conceptualization, investigation, visualization. **Mingzhen Su:** investigation, visualization, writing – review and editing, resources. **Klemens Budde:** writing – review and editing, conceptualization, supervision. **Mira Choi:** conceptualization, investigation, writing – original draft, writing – review and editing, supervision, methodology, formal analysis, project administration.

## Conflicts of Interest

The authors declare no conflicts of interest.

## Data Availability

Data sharing not applicable to this article as no datasets were generated or analysed during the current study.
